# Association of Alzheimer’s disease polygenic risk scores with amyloid accumulation in cognitively intact older adults

**DOI:** 10.1186/s13195-022-01079-4

**Published:** 2022-09-23

**Authors:** Emma S. Luckett, Yasmina Abakkouy, Mariska Reinartz, Katarzyna Adamczuk, Jolien Schaeverbeke, Sare Verstockt, Steffi De Meyer, Koen Van Laere, Patrick Dupont, Isabelle Cleynen, Rik Vandenberghe

**Affiliations:** 1 Laboratory for Cognitive Neurology, KU Leuven, Leuven, Belgium; 2 Alzheimer Research Centre KU Leuven, Leuven Brain Institute, Leuven, Belgium; 3 Laboratory for Complex Genetics, KU Leuven, Leuven, Belgium; 4 Bioclinica, Newark, CA USA; 5 Translational Research Center for GastroIntestinal Disorders, Department of Chronic Diseases, Metabolism and Ageing, KU Leuven, Leuven, Belgium; 6 Laboratory of Molecular Neurobiomarker Research, KU Leuven, Leuven, Belgium; 7 Division of Nuclear Medicine, UZ Leuven, Leuven, Belgium; 8 Nuclear Medicine and Molecular Imaging, Department of Imaging and Pathology, KU Leuven, Leuven, Belgium; 9 Neurology Department, University Hospitals Leuven, Herestraat 49, 3000 Leuven, Belgium

**Keywords:** Alzheimer’s disease, F-PACK, Polygenic risk score, Amyloid-PET, Longitudinal study

## Abstract

**Background:**

Early detection of individuals at risk for Alzheimer’s disease (AD) is highly important. Amyloid accumulation is an early pathological AD event, but the genetic association with known AD risk variants beyond the *APOE4* effect is largely unknown. We investigated the association between different AD polygenic risk scores (PRS) and amyloid accumulation in the Flemish Prevent AD Cohort KU Leuven (F-PACK).

**Methods:**

We calculated PRS with and without the *APOE* region in 90 cognitively healthy F-PACK participants (baseline age 67.8 (52–80) years, 41 *APOE4* carriers), with baseline and follow-up amyloid-PET (time interval 6.1 (3.4–10.9) years). Individuals were genotyped using Illumina GSA and imputed. PRS were calculated using three *p*-value thresholds (pT) for variant inclusion: 5 × 10^−8^, 1 × 10^−5^, and 0.1, based on the stage 1 summary statistics from Kunkle et al. (Nat Genet 51:414–30, 2019). Linear regression models determined if these PRS predicted amyloid accumulation.

**Results:**

A score based on PRS excluding the *APOE* region at pT = 5 × 10^−8^ plus the weighted sum of the two major *APOE* variants (rs429358 and rs7412) was significantly associated with amyloid accumulation (*p* = 0.0126). The two major *APOE* variants were also significantly associated with amyloid accumulation (*p* = 0.0496). The other PRS were not significant.

**Conclusions:**

Specific PRS are associated with amyloid accumulation in the asymptomatic phase of AD.

**Supplementary Information:**

The online version contains supplementary material available at 10.1186/s13195-022-01079-4.

## Background

Alzheimer’s disease (AD) is the most common form of dementia in the global population, with a complex interplay of both genetic and environmental factors [1]. Changes observed in AD, such as the accumulation of brain amyloid-β protein, can occur more than a decade prior to symptom onset (the preclinical or asymptomatic phase) [2]. Age is one of the largest risk factors for AD risk, where many of the observed pathological changes occur as age increases [3]. It is of high importance to develop methods that detect those individuals at risk of developing the disease prior to symptom onset, as this phase presents a window of opportunity for early disease diagnosis, treatment administration, and determining individuals suitable for prevention trials.

Apolipoprotein E *ε*4 (*APOE4*) is the largest genetic risk factor for development of sporadic AD [4] and has been shown to increase amyloid deposition in a dose-dependent manner, in the asymptomatic, the mild cognitive impairment and the dementia stages of AD [5–7]. Accumulation of brain amyloid in the asymptomatic phase occurs in a sigmoidal fashion, prior to reaching a plateau [8]. However, genetic risk for amyloid accumulation is largely unknown above the known effect of *APOE4*.

Beyond *APOE*, recent large-scale genome-wide association studies (GWAS) have highlighted low effect size variants in non-*APOE* genes that modify AD dementia risk [9–13]. In isolation, the identified risk SNPs have limited use in predicting AD risk. Combinations of selected AD risk SNP scores have previously been implemented in case-control studies, and have shown an increased risk of AD with a higher score (e.g. [14]). A polygenic risk score (PRS) approach to disease risk prediction is able to take into consideration all SNPs that show an association with AD, despite their small effect sizes, by capturing the overall genetic risk for an individual into a single score. This has already been performed in AD case-control studies (e.g. [14–16]), but there is a requirement for investigation in the asymptomatic phase, as well as determining the genetic risk for amyloid accumulation.

There are still many factors unknown about how to best model the PRS, for example the optimal threshold for SNP inclusion (pT) and how to represent the effect of *APOE* (e.g. for AD risk). A seminal study from Escott-Price et al. [15] suggested a PRS with the highest prediction accuracy (AUC = 78.2%) in predicting AD cases from controls is built using a liberal threshold for SNP inclusion (pT < 0.5), suggesting a polygenic architecture to AD. However, more recent studies have implied a more oligogenic architecture to AD, in which there are fewer SNPs associated with disease risk [17]. This suggests the use of more stringent (closer to genome-wide significant) pTs for the calculation of PRS for AD.

A more recent study from Leonenko et al. [18] further evaluated how best to model the *APOE4* effect in the context of PRS and found that the best prediction accuracy to predict AD cases from controls (AUC = 74.1%) came from a combination of a PRS excluding the *APOE* region with the addition of the weighted sum of two directly genotyped *APOE ε2* and *ε4* alleles (rs429358, rs7412, PRS_*noAPOE*_+*APOE*_*ε*2+*ε*4_) and a threshold pT < 0.1. This was when comparing PRS built using different SNP combinations, including PRS_*noAPOE*_ and PRS including all possible SNPs, at varying thresholds.

We aimed to evaluate PRS built as PRS_*noAPOE*_+*APOE*_*ε*2+*ε*4_, using different thresholds for SNP inclusion to determine the association with amyloid accumulation. We included cognitively intact older individuals participating in the Flemish Prevent AD Cohort KU Leuven (F-PACK), some of whom are in the asymptomatic stage of AD. We compared the performance of PRS_*noAPOE*_+*APOE*_*ε*2+*ε*4_ to other PRS.

## Methods

### Study participants

The Laboratory for Cognitive Neurology follows a cohort of 180 community-recruited deeply phenotyped older adults, known as F-PACK. F-PACK individuals were recruited between 2009 and 2015 in three waves of 60 individuals. At recruitment, individuals had to be aged between 50 and 80 years old, score ≥ 27 on the mini mental state examination (MMSE) and score zero on the clinical dementia rating (CDR) scale. Furthermore, individuals had to score within published norms on an extensive neuropsychological test battery [19, 20]. Exclusion criteria included a history of neurological or psychiatric illness, contraindication for magnetic resonance imaging (MRI), focal brain lesions on MRI, history of cancer, or exposure to radiation one year preceding the baseline amyloid positron emission tomography (PET) scan. Recruitment was stratified for two genetic factors: *APOE4* allele (present or absent) and *BDNF* status (*66 met* allele present or absent). This was carried out such that per 5-year age bin each factorial cell contained the same number of individuals matched for age, sex, and education. All recruited individuals received structural MRI and an ^18^F-Flutemetamol amyloid-PET scan at baseline. Participants are invited for 2-yearly neuropsychological assessments over a 10-year period.

F-PACK individuals received blood collection for genotyping at recruitment. A subset of 90 participants have additionally received a follow-up amyloid-PET scan, on average 6.1 (3.4–10.9) years after baseline.

The protocol was approved by the Ethics Committee University Hospitals Leuven. All participants provided written informed consent in accordance with the declaration of Helsinki.

### Structural MRI acquisition

At baseline and follow-up, each participant received a high resolution T1-weighted structural MRI scan for the PET processing procedure described below. Scans were performed using a 3T Philips Achieva dstream 32-channel headcoil MRI scanner (Philips, Best, The Netherlands). All baseline and 69 follow-up scans were acquired using the same 3D turbo field echo sequence: repetition time = 9.6 ms; echo time = 4.6 ms; flip angle = 8°; field of view = 250 × 250 mm; 182 slices; voxel size 0.98 × 0.98 × 1.2 mm^3^. Eighteen follow-up scans were acquired using a three-dimensional magnetisation-prepared rapid gradient-echo sequence, due to being acquired as part of The Amyloid Imaging to Prevent Alzheimer’s Disease study (AMYPAD): repetition time = 6.6 ms; echo time = 3.1 ms; flip angle = 9°; field of view = 270 × 252 mm; 170 slices; voxel size 1.05 × 1.05 × 1.2 mm^3^. Three individuals refused a follow-up scan.

### ^18^F-Flutemetamol PET acquisition and pre-processing

As previously described, ^18^F-Flutemetamol PET scans were acquired on a 16-slice Biograph PET/CT scanner (Siemens, Erlangen, Germany) at baseline and follow-up (6.1 (3.4–10.9) years), with a net injected intravenous dose of 149 MBq (127–162 MBq) and 156 MBq (77–198 MBq), respectively, with an acquisition window of 90–120 min post-injection [19, 21–24]. Four follow-up PET scans had an acquisition window of 90–110 min due to acquisition prior to a protocol amendment. A low-dose CT scan was acquired prior to all scans for attenuation correction. Random and scatter corrections were applied. Scans were reconstructed as frames of five minutes. All scans were reconstructed using ordered subsets expectation maximisation. All baseline scans and 73 follow-up scans were reconstructed as five iterations in eight subsets. Sixteen follow-up scans were reconstructed as four iterations in 21 subsets, due to being reconstructed as part of AMYPAD. The spatial resolution of the scanner is 4.6 mm full width at half maximum 1cm off centre measured with the NEMA protocol, and all scans were smoothed with a 5-mm full width at half maximum Gaussian filter.

### ^18^F-Flutemetamol image analysis

Statistical Parametric Mapping version 12 (SPM12, Wellcome Trust Centre for Neuroimaging, London, UK, http://www.fil.ionucl.ac.uk/spm) running on MATLAB R2018b (Mathworks, Natick, MA, USA) was used to process the images, as described previously [19, 21–24].

We calculated (mean) standardised uptake value ratios (SUVRs) from the spatially normalised images (voxel size: 2 × 2 × 2 mm^3^) in a composite cortical volume of interest (SUVR_*comp*_) based on the automated anatomical labelling atlas (AAL). This composite volume of interest included the following bilateral regions: frontal (AAL areas 3–10, 13–16, 23–28), parietal (AAL 57–70), anterior cingulate (AAL 31–32), posterior cingulate (AAL 35–36), and lateral temporal (AAL 81–82, 85–90) and was masked with the participant-specific grey matter (GM) segmentation map (threshold = 0.3) [19, 25]. Cerebellar grey matter was used as the reference region to calculate SUVR_*comp*_, defined as AAL areas 91–108 and was masked by the participant-specific GM map (intensity threshold = 0.3) [19]. SUVR_*comp*_ were then converted to Centiloids (CL) using the formula CL = 127.6 × SUVR_*comp*_–149 [26, 27]. Individuals were deemed amyloid positive when their CL was ≥ 23.5, a pathologically confirmed cut-off for amyloid positivity [28]. Furthermore, to model amyloid change longitudinally, we calculated rate of change as:

$$Amyloid\ rate\ of\ change=\frac{Follow- up\ Centiloid- Baseline\ Centiloid}{Time\ interval\ (years)}$$

For use in secondary analyses, we repeated the PET image processing pipeline including a partial volume correction (PVC, 5 mm full-width half-maximum) step, using a modified Müller-Gärtner procedure [29]. Rate of change was derived, as per the above equation using SUVRs, of these corrected images for use in the analyses described below.

### Structural MRI longitudinal change

In a secondary analysis, we also examined whether any PRS with significant predictive value for amyloid accumulation also had any predictive value for longitudinal MRI volume loss. As a measure of atrophy, we used the GM maps at baseline and follow-up, produced from the PET processing procedure, to calculate the percental whole brain GM volume change between the two time points as:

$$\frac{Grey\ matter\ volume\ at\ baseline- Grey\ matter\ volume\ at\ follow- up\ }{Grey\ matter\ volume\ at\ baseline}\ x\ 100$$

Furthermore, for a voxelwise analysis we calculated GM change images, as follow-up GM map minus baseline GM map, using SPM12, for use in voxelwise regression analyses described below.

### Modelling of longitudinal cognitive change

For the PRS with predictive value for amyloid accumulation, we also examined whether this predictive power extended to cognitive change. To model cognitive change, based on a prior study [20], the mean Buschke Selective Reminding Test Total Retention (BSRT TR) score [30] was selected and slopes were calculated using latent growth curve analysis, using the R package *Lavaan* [31]. Individuals are being followed for a period of 10 years with two-yearly neuropsychological evaluations, so we used mean BSRT TR scores from all available neuropsychological testing time points (FUY) up until the closest to the follow-up amyloid-PET scan (number of individuals at each FUY: 4 FUY2, 37 FUY4, 32 FUY6, 10 FUY8, 7 FUY10; mean interval between follow-up amyloid-PET and closest neuropsychological assessment is 13.06 ± 12.04 months). Missing values were imputed in R using the CART imputation method in the package *mice* [32]. The calculated mean BSRT TR slopes were then used further, where a more negative slope represents steeper decline.

### Genetic data acquisition and processing

DNA was further available for 177 F-PACK participants, which was subsequently genotyped using the Illumina Global Screening Array (GSA, coverage of 657,598 SNPS) in collaboration with the Institute of Clinical and Medical Biology (University Hospital Schleswig-Holstein, Germany) [33]. Standard quality control (QC) was performed using PLINK (Version 1.9, www.cog-genomics.org/plink/1.9) and included SNP call rate ≥ 0.95, minor allele frequency (MAF) ≥ 0.01, and outlying heterozygosity (± 5 standard deviations) [34]. Hardy-Weinberg equilibrium threshold = 1 × 10^−6^ was also applied. Ethnic outliers were detected using the Phase 3 1000 Genomes (1KG) dataset (*N* = 2504 [35]). Imputation was performed using the Michigan Imputation Server (https://imputationserver.sph.umich.edu) [36] and Haplotype Reference Consortium reference panel (http://www.haplotype-reference-consortium.org), resulting in 39,131,578 SNPs. Data were filtered with imputation information score > 0.7 and MAF ≥ 0.01. After imputation and QC 7,466,483 SNPs remained for further analysis. Three individuals were removed during the QC process (one due to sample duplication, one due to relatedness (pi-hat > 0.2), and one due to outlying heterozygosity).

### Polygenic risk score calculation

PRS calculations were performed using PRSice-2 [37]. The stage 1 summary statistics from the GWAS performed by Kunkle et al. [11] were used as the base file, and the European individuals from 1KG (*N* = 503 [35]) were used as an external reference panel for clumping to remove SNPs in high linkage disequilibrium (clumping window = 250 kilobases, *r*^2^ = 0.1). Post-clumping, there were 335,326 SNPs remaining for PRS calculations. We first calculated PRS using the PRS_*noAPOE*_+*APOE*_*ε*2+*ε*4_ approach [18]. We calculated PRS excluding the *APOE* region (chromosome 19: 45-48.8 Mb, PRS_*noAPOE*_) at three thresholds for SNP inclusion (pT): pT = 0.1; 1 × 10^−5^; 5 × 10^−8^, and then added the weighted sum of the two major *APOE* SNPs (rs429358 and rs7412, *APOE*_*ε*2+*ε*4_), using the effect sizes from Kunkle et al. [11], to each score (N1 * β1 + N2 * β2, where N1 and N2 are the number of alleles for each *APOE* SNP, respectively, and β1 and β2 are the corresponding effect sizes).

For comparison, we also calculated PRS_*AD*_, in which all available SNPs were included at each pT, as well as PRS_*APOEonly*_, in which PRS were calculated at each pT using only the *APOE* region specified above. All PRS were *z*-score normalised prior to further analyses.

### Statistical analyses

Statistical analyses were performed in R version 4.1.0 (2021-05-18; The R Foundation for Statistical Computing; https://cran.r-project.org/). Prior to analyses, Shapiro-Wilk tests were used to determine data normality.

Cohort characteristics were assessed between *APOE4* carriers and non-carriers using Wilcoxon rank sum tests with continuity correction or Welch two-sample *t*-tests for continuous data, depending on normality, and *χ*^2^ tests for categorical data.

### Primary analyses

For our primary analysis, linear regressions were performed for each PRS_*noAPOE*_+*APOE*_*ε*2+*ε*4_ (i.e. at 3 different SNP inclusion thresholds) as predictor, and amyloid rate of change as outcome variable. Baseline age, sex, and the first three principal components were included as covariates. Inference was based on an uncorrected *p* < 0.05 threshold divided by the number of SNP inclusion thresholds (*N* = 3).

### Secondary analyses

We investigated how PRS_*noAPOE*_+*APOE*_*ε*2+*ε*4_ performed compared to other PRS, thus linear regressions were also performed as described above with PRS_*AD*_, *APOE*_*ε*2+*ε*4_, PRS_*noAPOE*_, or with PRS_*APOEonly*_. Per type of PRS, inference was based on an uncorrected *p* < 0.05 threshold divided by the number of SNP inclusion thresholds (*N* = 3), except for *APOE*_*ε*2+*ε*4_ given that this score does not rely on *p*-value thresholds for SNP inclusion as it is built using only the weighted sum of two *APOE* SNPs. We also investigated whether all calculated PRS were associated with baseline amyloid burden using the same statistical approach.

In a further secondary analysis, we determined the ability for any significant PRS to discriminate individuals who were amyloid negative at both time points, on the one hand, from individuals who were amyloid negative at baseline and positive at follow-up or amyloid positive at both time points, on the other hand. We performed a set of receiver operating characteristic (ROC) analyses using the R package *pROC* based on a logistic regression classifier [38]. We included two demographic models (one model with age + sex; one model with age + sex + *APOE4* status (yes/no)) and models including any significant PRS determined from the previous analyses, with age and sex. Measures of performance are given as areas under the curve (AUCs) and 95% confidence intervals (CI). Pairwise comparisons of model AUCs were performed using the DeLong method [39].

To evaluate the effect of PVC, we also carried out the regression analyses with the longitudinal change in PVC SUVRs as predictor.

Finally, we performed the regression analyses for cognitive decline or GM atrophy, with any PRS that had a significant association with amyloid rate of change. In addition, we performed whole-brain voxelwise *t*-tests using the GM change maps, with the significant PRS and time interval as covariates. We used a FWE corrected cluster-level threshold of *p* < 0.05 with the voxel-level set at uncorrected *p* < 0.001 [40].

## Results

### F-PACK characteristics

**Fig. 1:**
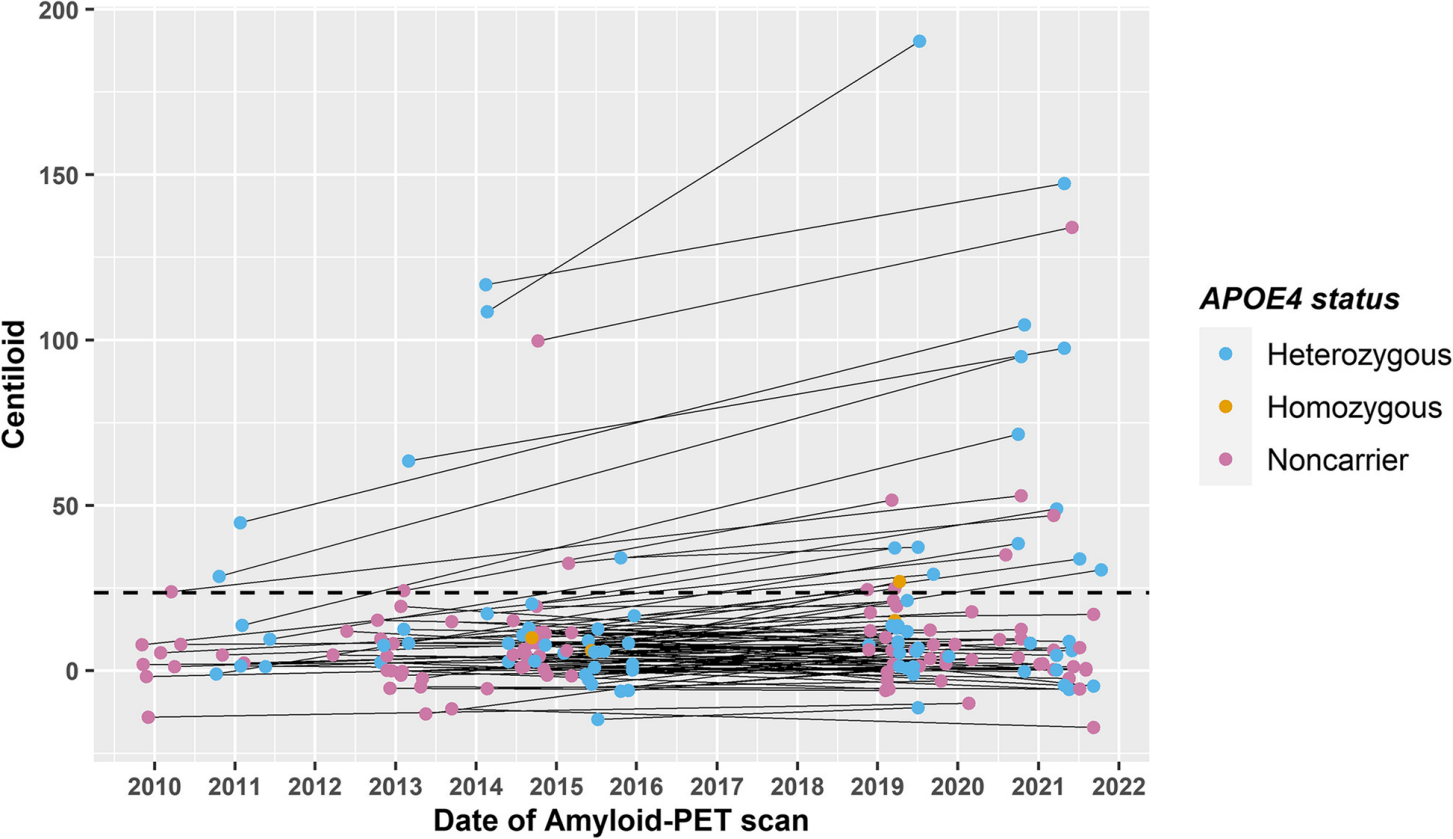
Change in amyloid load between baseline and follow-up for F-PACK participants, expressed in Centiloids. The dotted line represents the threshold for amyloid positivity ≥ 23.5 [28]. *N* = 90

From the 90 F-PACK individuals with follow-up amyloid-PET included in this study, nine were amyloid positive at baseline (10%) and 21 at follow-up (23%, Table 1, Fig. 1). *APOE4* carriers had a significantly higher amyloid rate of change (rate of change for *APOE4* carriers: 1.06 (range: − 2.85–13.92); *APOE4* non-carriers: − 0.03 (range: − 3.03–4.22) *p* = 0.01, outlier excluded *p* = 0.02) and a significantly lower time interval between amyloid-PET scans than non-carriers (time interval for *APOE4* carriers: 5.4 (range: 3.4–10.0) years; *APOE4* non-carriers 6.1 (range: 4.0–10.9) years, *p* = 0.008). There was also a significantly higher number of *APOE4* carriers classified as amyloid positive at follow-up (34%) compared to non-carriers (14%, *p* = 0.049). Two individuals had a CDR that had evolved to 0.5 at the time of follow-up amyloid-PET.

**Table 1:** Baseline F-PACK characteristics stratified for *APOE4* polymorphism status for individuals with baseline and follow-up amyloid-PET. Data are reported as median and range (minimum to maximum) for continuous variables and numerical for categorical variables. Wilcoxon rank sum test with continuity correction or Welch two-sample *t*-tests were used for continuous data, depending on data normality. *χ*^2^ tests have been used for categorical data. *ε*2*ε*3 *N* = 7; *ε*2*ε*4 *N* = 2; *ε*3*ε*3 *N* = 42; *ε*3*ε*4 *N* = 37; *ε*4*ε*4 *N* = 2. Total *N* = 90

	***APOE4*** non-carrier (***n***=49)	***APOE4*** carrier (***n***=41)	Statistics
**Sex (male/female)**	24/25	22/19	*Χ*^*2*^ = 0.05, *p* = 0.82
**BDNF** ***66 met*** **carriers**	24	23	*Χ*^*2*^ = 0.21, *p* = 0.64
**Age (years)**	67 (52–80)	68 (56–79)	*T* = –0.18, *p* = 0.86
**Education (years)**	14 (8–20)	16 (9–23.5)	*T* = 1.76, *p* = 0.08
**MMSE**	29 (27–30)	29 (27–30)	*W* = 1022.5, *p* = 0.88
**CDR**	0	0	*NA*
**AVLT TL (/75)**	47 (30–69)	46 (35–68)	*T* = − 0.16, *p* = 0.87
**AVLT %DR**	85.7 (30–107.7)	86.7 (58.3–107.7)	*W* = 1039.5, *p* = 0.78
**BSRT TR (/12)**	8.2 (5.6–10.8)	7.9 (4.9–10.5)	*T* = − 0.94, *p* = 0.35
**BSRT DR (/12)**	8 (2–12)	8 (3–12)	*W* = 932.5, *p* = 0.56
**BNT (/60)**	57 (46–60)	57 (41–60)	*W* = 1028.5, *p* = 0.85
**AVF (# words)**	23 (14–40)	23 (14–42)	*T* = 0.50, *p* = 0.62
**LVF (# words)**	36 (14–65)	37 (9–61)	*T* = − 0.05, *p* = 0.96
**PALPA49 (/30)**	28 (20–30)	27 (23–30)	*W* = 981.5, *p* = 0.85
**RPM (/60)**	46 (22–57)	45 (22–57)	*W* = 864, *p* = 0.26
**TMT B/A**	2.2 (1.2–4.8)	2.4 (1.0–4.8)	*T* = 0.69, *p* = 0.49
**Baseline Centiloid**	4.8 (− 14.1–99.8)	7.7 (− 14.8–116.8)	*W* = 1163, *p* = 0.20
**Baseline amyloid positivity**	4 (8%)	5 (12%)	*Χ*^*2*^ = 0.08, *p* = 0.78
**Follow-up amyloid positivity**	7 (14%)	14 (34%)	*Χ*^*2*^ = 3.87, *p* = 0.049
**Amyloid rate of change**	− 0.03 (− 3.03–4.22)	1.06 (− 2.85–13.92)	*W* = 1313, *p* = 0.01
**Time interval (years)**	6.1 (4.0–10.9)	5.4 (3.4–10.0)	*W* = 675, *p* = 0.008

*Abbreviations*: *AVF* Animal Verbal Fluency Test, *AVLT TL/DR* Rey Auditory Verbal Learning Test Total Learning/Delayed Recall, *BNT* Boston Naming Test, *BSRT TR/DR* Buschke Selective Reminding Test Total Retention/Delayed Recall, *CDR* Clinical Dementia Rating scale, *LVF* Letter Verbal Fluency Test, *MMSE* Mini Mental State Examination, *PALPA49* Psycholinguistic Assessment of Language Processing in Aphasia (PALPA) subtest 49, *RPM* Raven’s Progressive Matrices, *TMT B/A* Trail Making Test part B divided by part A

There was an outlier with amyloid rate of change (as determined by Grubb’s test in R, using the package *Outliers*). Therefore, the amyloid analyses were repeated excluding this individual.

### Primary analyses: PRS_*noAPOE*_+*APOE*_*ε*2+*ε*4_

**Fig. 2:**
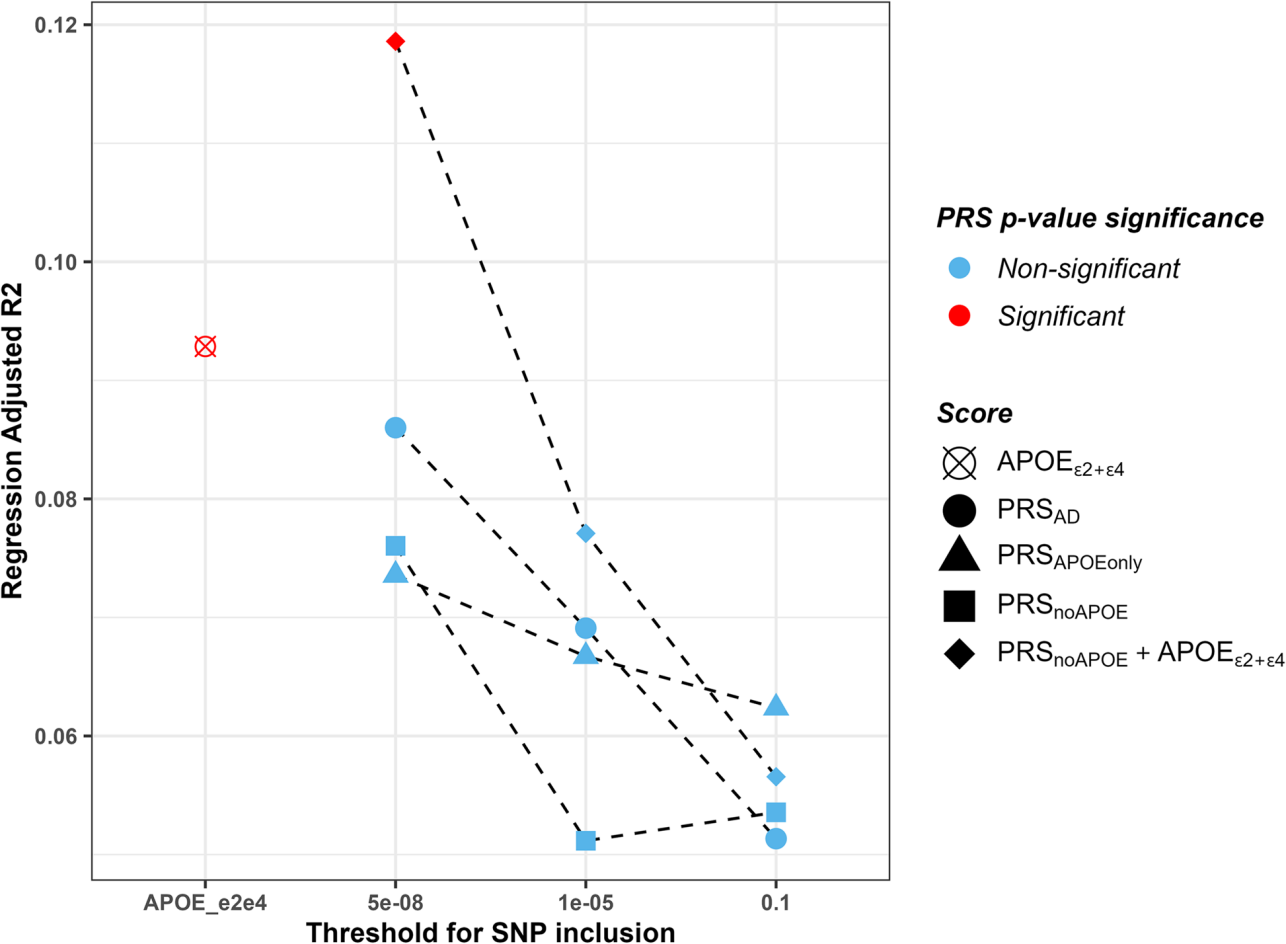
The adjusted *R*^2^ and PRS *p*-values for the regression models. The red points represent significant PRS *p*-values from the regression models (< 0.017 (multiple pT), or < 0.05 (for single score)). The higher the adjusted *R*^2^ the higher the variance explained by the PRS on amyloid rate of change in the linear regression model. *N* = 90

**Fig. 3:**
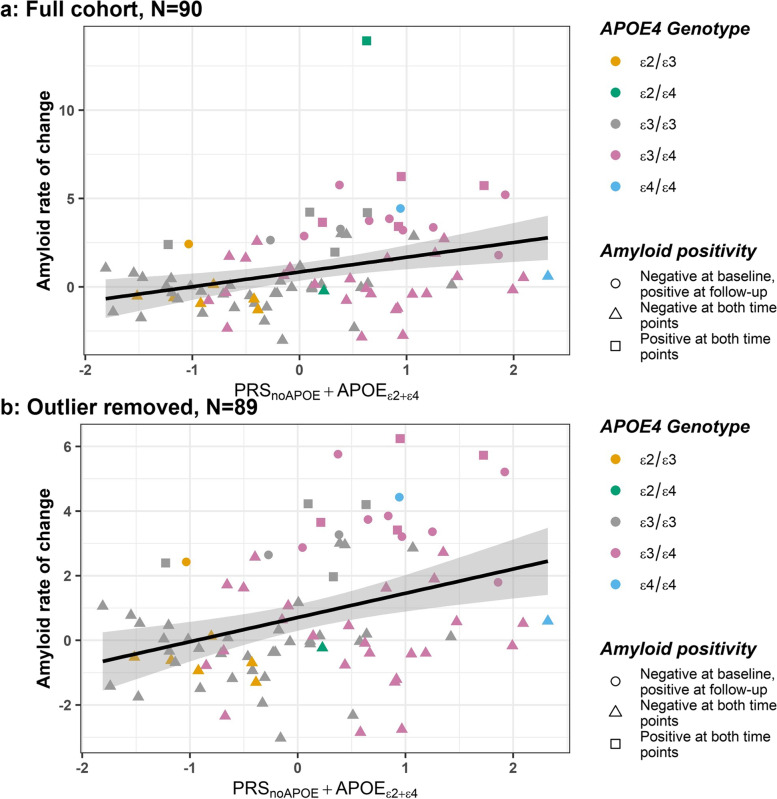
Regression plots for PRS_*noAPOE*_+*APOE*_*ε*2+*ε*4_ at pT = 5 × 10^−8^ and amyloid rate of change. **A** For the whole cohort. *N* = 90. **B** When the outlier for amyloid rate of change is removed. *N* = 89. The shape indicates amyloid status based on CL ≥ 23.5 [27]: amyloid negative at both time points (triangle, *N* = 69); amyloid negative at baseline and amyloid positive at follow-up (circle, *N* = 12); and amyloid positive at both time points (square, *N* = 9). Amyloid rate of change is follow-up Centiloid minus baseline Centiloid, divided by the time interval (years). Participants are coloured based on *APOE4* genotype (*ε*2*ε*3 *N* = 7; *ε*2*ε*4 *N* = 2; *ε*3*ε*3 *N* = 42; *ε*3*ε*4 *N* = 37; *ε*4*ε*4 *N* = 2; outlier is *ε*2*ε*4)

PRS_*noAPOE*_+*APOE*_*ε*2+*ε*4_ had a significant association with amyloid rate of change when the SNP inclusion threshold (pT) = 5 × 10^−8^ (*p* = 0.0126, *β* = 0.68 (95% CI 0.15, 1.20), *R*^2^ = 0.12, Figs. 2 and 3A, Table 2). When the outlier was removed, significance remained (*p* = 0.004, *β* = 0.65 (95% CI 0.21, 1.09), *R*^2^ = 0.11, Fig. 3B). Figure 2 shows that the highest *R*^2^ and lowest *p*-value occur when the pT is more stringent.

**Table 2:** Regression results for the different PRS and amyloid rate of change. Raw values are shown, significant *p*-values in bold (< 0.017 (multiple pT) or < 0.05 (for single score)). *APOE* region: chromosome 19: 45–48.8 Mb. *APOE* SNPs: rs429358, rs7412. Baseline age, sex, and PCs 1–3 included as covariates. *N* = 90

Score	Score description	pT	Number of SNPs	Adjusted ***R***^**2**^	PRS ***p***-value	PRS β (95% CI)
PRS_*noAPOE*_+*APOE*_*ε*2+*ε*4_	All available SNPs at each pT excluding the *APOE* region, plus the weighted sum of the two major *APOE* SNPs	5 × 10^−8^	22	0.12	**0.0126**	0.68 (0.15–1.20)
		1 × 10^−5^	70	0.08	0.1188	0.45 (− 0.12–1.02)
		0.1	77,270	0.06	0.4319	0.21 (− 0.32–0.74)
PRS_*AD*_	All available SNPs at each pT	5 × 10^−8^	65	0.09	0.0721	0.48 (− 0.04–1.01)
		1 × 10^−5^	129	0.07	0.1895	0.36 (− 0.18–0.89)
		0.1	77,378	0.05	0.6874	− 0.10 (− 0.61–0.40)
*APOE*_*ε*2+*ε*4_	Weighted sum of the two major *APOE* SNPs		2	0.09	**0.0496**	0.55 (0.0009–1.09)
PRS_*noAPOE*_	All available SNPs at each pT excluding the *APOE* region	5 × 10^−8^	20	0.08	0.1262	0.37 (− 0.10–0.84)
		1 × 10^−5^	68	0.05	0.7016	0.10 (− 0.43–0.64)
		0.1	77,268	0.05	0.5510	− 0.15 (− 0.66–0.35)
PRS_*APOEonly*_	All available SNPs within the *APOE* region at each pT	5 × 10^−8^	46	0.07	0.1455	0.39 (− 0.14–0.91)
		1 × 10^−5^	62	0.07	0.2191	0.33 (− 0.20–0.86)
		0.1	118	0.06	0.2885	0.29 (−0.25–0.82)

Both regression plots in Fig. 3A and B highlight increasing amyloid rate of change with increasing PRS. One can also appreciate the lower PRS are for those participants with *ε*2*ε*3 or *ε*3*ε*3 *APOE* genotypes, whereas, in general, those participants with higher PRS carry at least one *ε*4 allele. No significance was found at the other pTs.

### Secondary analyses: other PRS models and phenotypes

From our secondary analyses, there was a significant association between amyloid rate of change and *APOE*_*ε*2+*ε*4_ (*p* = 0.0496, *β* = 0.56 (95% CI 0.0009, 1.09), *R*^2^ = 0.09, Fig. 2, Table 2). When the outlier was removed, the significance remained (*p* = 0.0201, *β* = 0.56 (95% CI 0.09-1.00), *R*^2^ = 0.08). None of the other PRS were significant with the total group. The results from the total cohort can be observed in Fig. 2, where red points represent significant *p*-values, and blue points are non-significant. From the figure, one can also appreciate the higher variance explained by the PRS on amyloid accumulation in the regressions when using the more stringent SNP inclusion thresholds. Of note is the higher *R*^2^ with PRS_*noAPOE*_+*APOE*_*ε*2+*ε*4_ when pT = 5 × 10^−8^ (*R*^2^ = 0.12) compared to when using *APOE*_*e*2+*e*4_ alone (*R*^2^ = 0.09). No PRS were associated with baseline amyloid load (Supplementary Table 1).

After performing PVC, the *p*-value for PRS_*noAPOE*_+*APOE*_*ε*2+*ε*4_ at pT = 5 × 10^−8^ was comparable to that obtained without PVC (values for PVC SUVRs: *p* = 0.0196 (*β* = 0.008 (95% CI 0.001, 0.014), *R*^2^ = 0.13). Note that we used SUVRs for the PVC analysis; hence, this has to be compared with the same analysis for the primary analysis using SUVRs as well. At pT = 5 × 10^−8^, the regression output for the primary analysis with non-PVC SUVRs is *p* = 0.0131 (*β* = 0.005 (95% CI 0.001, 0.009), *R*^2^ = 0.11). Significance was lost for *APOE*_*ε*2+*ε*4_ (*p* = 0.30, *β* = 0.005 (95% CI − 0.002, 0.011), *R*^2^ = 0.10) after PVC correction. For comparison, the regression output with SUVRs for *APOE*_*ε*2+*ε*4_ correspond to: *p* = 0.051, *β* = 0.004 (95% CI − 0.00002, 0.009), *R*^2^ = 0.08).

**Fig. 4:**
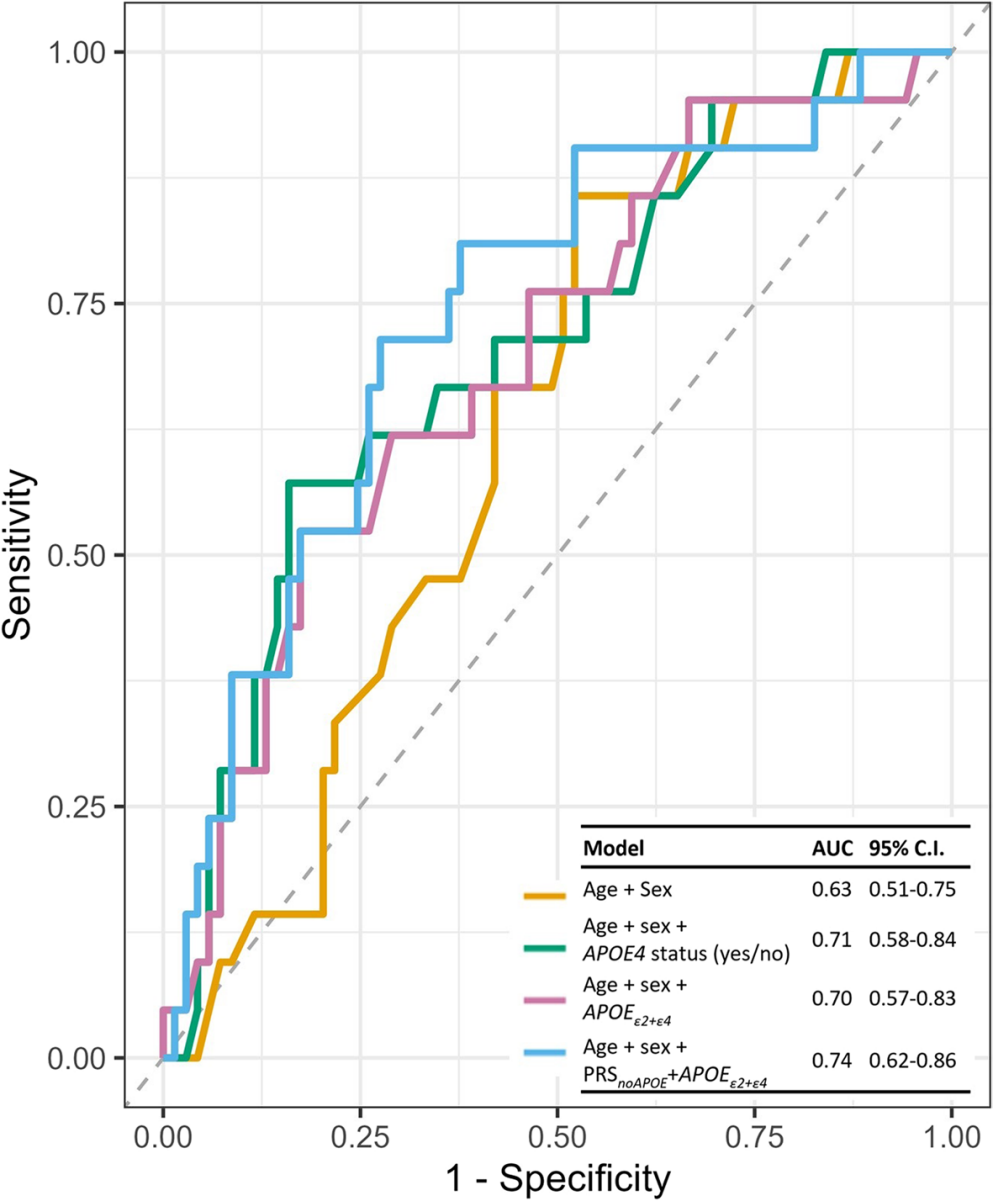
ROC curves for predicting amyloid status. PRS_*noAPOE*_+*APOE*_*ε*2+*ε*4_ (with pT = 5 × 10^−8^) with age and sex was numerically the best performing model at predicting individuals who were amyloid negative at both time points, on the one hand, from individuals who were amyloid negative at baseline and positive at follow-up or amyloid positive at both time points, on the other hand. Abbreviations: AUC, area under the curve; CI, confidence interval; ROC, receiver operating characteristic

Figure 4 shows the results from the ROC analysis. We determined the ability of PRS_*noAPOE*_+*APOE*_*ε*2+*ε*4_ and of *APOE*_*ε*2+*ε*4_ to discriminate amyloid negative individuals at both time points, on the one hand, from individuals who were amyloid negative at baseline and positive at follow-up or amyloid positive at both time points, on the other hand. The best performing model was age + sex + PRS_*noAPOE*_+*APOE*_*ε*2+*ε*4_ with pT = 5 × 10^−8^ (AUC = 0.74, 95% CI = 0.62–0.86). Numerically, the second best performing model was the demographic model consisting of age + sex + *APOE4* status (yes/no) (AUC = 0.71, 95% CI = 0.58–0.84). This had a similar performance to the model with age + sex + *APOE*_*ε*2+*ε*4_ (AUC = 0.70, 95% CI = 0.57–0.83). The demographic model including age + sex (AUC = 0.63, 95% CI = 0.51–0.75) performed the worst. None of the models were significantly different from each other when performing pairwise model comparisons (Supplementary Table 2).

PRS_*noAPOE*_+*APOE*_*ε*2+*ε*4_ and *APOE*_*ε*2+*ε*4_ were not associated with cognitive decline over the time course measured. Neither were these PRS associated with longitudinal whole-brain grey matter atrophy nor were there significant effects based on the whole-brain voxelwise analyses (Supplementary Table 3).

## Discussion

Our study has highlighted that specific AD PRS are associated with amyloid accumulation in the asymptomatic phase of the disease, when built using a stringent SNP inclusion threshold.

The present study primarily investigated the association of amyloid change and PRS_*noAPOE*_+*APOE*_*ε*2+*ε*4_, given PRS built in this way have recently been shown to give the best prediction accuracy to predict AD cases from controls (AUC = 74.1%) with pT < 0.1 [18]. In this previous paper, this PRS was superior at predicting AD cases from controls, compared to PRS built using different SNP combinations, including PRS_*noAPOE*_ and PRS_*AD*_, at varying thresholds. In that study, the best prediction accuracy for PRS_*AD*_ reached an AUC of 69.8% (pT = 5 × 10^−8^) and PRS_*noAPOE*_ AUC = 61.3% (pT = 0.1). Furthermore, these were not better performing than the two major *APOE* SNPs (rs429358 and rs7412) alone (AUC = 70.0%) [18]. The data presented in the present study also suggest that PRS_*noAPOE*_+*APOE*_*ε*2+*ε*4_ with pT = 5 × 10^−8^ is superior than the other PRS at having an association with amyloid accumulation in the asymptomatic phase of AD. The effect sizes observed for this score are higher than those observed in the other models. This score had a more significant association with amyloid accumulation than the major *APOE* SNPs, in the linear regressions, and produced, numerically, the highest AUC (AUC = 74%) compared to the demographic models (AUC for age + sex = 63% and AUC for age + sex + *APOE4* status (yes/no) = 71%) and the model including the weighted sum of the two major *APOE* SNPs (AUC = 70%). The AUC of 74% is comparable to those reported in the literature in AD case-control prediction studies. This score also had a borderline significant association with amyloid rate of change after PVC was performed. The slight loss in significance is likely due to the introduction of additional noise from the PVC. This can occur when PVC is performed on PET scans of cognitively intact older adults, which pertains to the F-PACK cohort [41]. However, the results further strengthen the hypothesis that there are other variants above *APOE* that are important for amyloidogenic processes. The increasing levels of amyloid in this asymptomatic phase may be associated with these lower effect size variants, whereas in the latter disease stages these associations are absent due to amyloid accumulation reaching a plateau [8].

This study investigated whether the thresholds for SNP inclusion that were found to be optimal in AD case-control studies were also applicable in the asymptomatic phase to detect amyloid changes. The results show stringent thresholds for SNP inclusion have a significant association with amyloid accumulation compared to the more liberal threshold of pT = 0.1. More liberal thresholds may be optimal for predicting cases from controls, but the present study suggests when trying to detect amyloid accumulation in asymptomatic AD, stringent thresholds for SNP inclusion, that reduce noise, are more optimal.

Previous studies have been unable to demonstrate an association between PRS and amyloid accumulation (e.g. [42, 43]). It is consistently found in many studies that *APOE4* is associated with higher amyloid load and amyloid accumulation (in the asymptomatic phase [6, 44]), but the PRS-based studies were unable to provide evidence for an association between PRS and baseline amyloid nor PRS and amyloid accumulation. Consistent with previous studies, the data from the present study replicate the effect of *APOE4* on amyloid accumulation, as well as providing a lack of association between PRS and baseline amyloid load. However, the present study did find an association between a specific PRS and amyloid accumulation over time. It can be observed that some individuals in the F-PACK cohort exhibit negative rates of change, along with the individuals that increase. Figure 1 shows the change in amyloid over time for the F-PACK cohort. The range in amyloid load below the threshold is narrow, whereas the change in amyloid above threshold is much larger, highlighting that the most change is driven by changes above the threshold of amyloid positivity. This change is not occurring below the threshold and change is thus minimal, even with those participants with a negative amyloid rate of change. Furthermore, a sensitivity analysis was conducted, whereby we removed the individuals in whom amyloid increased but did not surpass the threshold for positivity, and the regression still produced significant results with pT = 5 × 10^−8^ (data not shown due to the introduction of potential bias from removing data a posteriori). However, this further strengthens the primary analysis results that the observed effect is not driven by spurious accumulations in amyloid. Thus, the use of PRS to predict amyloid accumulation in the asymptomatic phase allows for testing genetic determinants of the amyloidogenic processes in sporadic AD, in the absence of downstream secondary effects in this early disease stage.

The effect of the *APOE* variants is clearly high given the results presented. This highlights that in the asymptomatic stage *APOE4* has a large influence on the association of a PRS with amyloid change. When the threshold for SNP inclusion is more than genome-wide significant (> 5 × 10^−8^), the variance explained decreases, which is coupled with a loss in significance of the PRS. Thus, lower effect size variants play a role in AD risk, but increasing the threshold for SNP inclusion beyond genome-wide significance results in the addition of SNPs that create noise, thus suggesting an oligogenic architecture to (asymptomatic) AD. Nevertheless, the variance explained by the PRS on amyloid accumulation is low, thus there may be a large influence from other factors, such as gene-environment interactions.

### Limitations

Some limitations need to be considered. The amyloid scans were acquired between 90 and 120 min post injection, and modelling of cerebral blood flow (CBF) changes over time was therefore not possible. According to a cross-sectional study in the Baltimore Longitudinal Study of Aging, amyloid SUVR can be influenced by CBF and mostly so in individuals with amyloid PET values in the upper tertile [45]. The effect of CBF on amyloid PET SUVR has been further quantified in a simulation study [46]. The increases in CBF that are needed to account for a 1% change in SUVR is of the order of 5–15% increase in CBF, and higher CBF changes are needed to affect SUVR when amyloid load is lower ([46], Fig. 3). In a cognitively normal longitudinal cohort of individuals who remain relatively stable on a cognitive level and who do not manifest a neurological disease, a longitudinal CBF change of that order is implausible, also given the strict regulation of CBF under physiological circumstances. Note that CBF would need to increase over time with higher PRS scores. This can only be empirically excluded by concomitant longitudinal blood flow studies. The time interval between the baseline and follow-up amyloid-PET scans was variable, some individuals with a shorter interval than others. Amyloid accumulation is not a linear process; therefore, a difference in the onset of the rising phase of amyloid accumulation as well as the slope may be driving the differences we observe. The PRS were built using GWAS data from Kunkle et al. [11] that are not necessarily transferable to other ethnicities; thus, results should be carefully considered when inferring associations.

## Conclusions

To conclude, a PRS built as PRS_*noAPOE*_+*APOE*_*ε*2+*ε*4_ with a stringent threshold for SNP inclusion had a more significant association with amyloid accumulation than the major *APOE* variants alone or than PRS built with other SNP combinations. This suggests an oligogenic, rather than polygenic, architecture to (asymptomatic) AD, in line with recent publications. The results may aid in participant recruitment and stratification for clinical trials, by identifying those individuals who are more susceptible to early brain amyloid changes, and thus more at risk to developing AD. According to the current dataset, PRS_*noAPOE*_+*APOE*_*ε*2+*ε*4_ outperforms the simple use of the *APOE4* polymorphism alone for this purpose.

## Supplementary Information

**Additional file 1: Supplementary Table 1.** Regression results for PRS with baseline amyloid load. *APOE* SNPs: rs429358, rs7412. Baseline age, sex and PCs 1-3 included as covariates. *N* = 90. **Supplementary Table 2.** Model comparisons between Receiver Operating Characteristic Areas Under the Curve using the DeLong method. **Supplementary Table 3.** Regression results for the significant PRS with cognitive decline or grey matter atrophy. Raw values are shown. *APOE* SNPs: rs429358, rs7412. Baseline age, sex and PCs 1-3 included as covariates. *N* = 90.

## Data Availability

Data are available upon reasonable request.

## Abbreviations

AAL: Automated anatomical labelling atlas
AD: Alzheimer’s disease
APOE4: Apolipoprotein E *ε*4
AUC: Area under the curve
AVF: Animal Verbal Fluency
AVLT: Rey’s Auditory Verbal Learning Test
BDNF: Brain-derived neurotrophic factor
BNT: Boston Naming Test
BSRT TR: Buschke Selective Reminding Test Total Retention
CBF: Cerebral blood flow
CI: Confidence interval
CDR: Clinical Dementia Rating
CGM: Cerebellar grey matter
CL: Centiloid
F-PACK: Flemish Prevent AD Cohort KU Leuven
FUY: Follow-up year neuropsychological testing time point
GM: Grey matter
GSA: Global Screening Array
GWAS: Genome-Wide Association Study
LVF: Letter Verbal Fluency
MAF: Minor allele frequency
MMSE: Mini Mental State Examination
MRI: Magnetic resonance imaging
PALPA49: Psycholinguistic Assessment of Language Processing in Aphasia item 49
PC: Principal component
PET: Positron emission tomography
PRS: Polygenic risk score
pT: *p*-value threshold for single nucleotide polymorphism inclusion
PVC: Partial volume correction
QC: Quality control
ROC: Receiver operating characteristic
RPM: Raven’s Progressive Matrices
SNP: Single nucleotide polymorphism
SUVR: Standardised uptake value ratio
SUVR_*comp*_: Standardised uptake value ratio in the composite cortical volume of interest
TMT: Trial Making Test
1KG: 1000 Genomes
