# Supplementary material for: Association of Alzheimer’s disease polygenic risk scores with amyloid accumulation in cognitively intact older adults
Source: Alzheimers Res Ther. 2022 Sep 23;14:138. doi: 10.1186/s13195-022-01079-4 (PMC9508733; doi:10.1186/s13195-022-01079-4)
Supplement: Supplementary file 1 — Additional file 1: Supplementary Table 1. Regression results for PRS with baseline amyloid load. APOE SNPs: rs429358, rs7412. Baseline age, sex and PCs 1-3 included as covariates. N = 90. Supplementary Table 2. Model comparisons between Receiver Operating Characteristic Areas Under the Curve using the DeLong method. Supplementary Table 3. Regression results for the significant PRS with cognitive decline or grey matter atrophy. Raw values are shown. APOE SNPs: rs429358, rs7412. Baseline age, sex and PCs 1-3 included as covariates. N = 90. [file 13195_2022_1079_MOESM1_ESM.docx]

**Supplementary tables**

**Supplementary Table 1.** Regression results for PRS with baseline amyloid load. *APOE* SNPs: rs429358, rs7412. Baseline age, sex and PCs 1-3 included as covariates. N = 90.

| **Score** | **Score description** | **pT** | **Number**  **of SNPs** | **Adjusted R^2^** | **PRS**  ***p*-value** | **PRS β**  **(95% CI)** |
| --- | --- | --- | --- | --- | --- | --- |
| PRS*_noAPOE_*+*APOE_ε_*_2+_*_ε_*_4_ | All available SNPs at each pT excluding the *APOE* region, plus the weighted sum of the two major *APOE* SNPs | 5x10^−8^ | 22 | 0.02 | 0.31 | 2.56  (-2.46-7.58) |
|  |  | 1x10^−5^ | 70 | 0.008 | 0.71 | 0.99  (-4.32-6.30) |
|  |  | 0.1 | 77,270 | 0.009 | 0.61 | 1.25  (-3.63-6.13) |
| PRS*_AD_* | All available SNPs at each pT | 5x10^−8^ | 65 | 0.04 | 0.09 | 4.21  (-0.67-9.10) |
|  |  | 1x10^−5^ | 129 | 0.03 | 0.17 | 3.43  (-0.52- 8.38) |
|  |  | 0.1 | 77,378 | 0.008 | 0.70 | 0.92  (-3.76-5.60) |
| *APOE_ε_*_2+_*_ε_*_4_ | Weighted sum of the two major *APOE* SNPs |  | 2 | 0.01 | 0.53 | 1.64  (-3.50-6.77) |
| PRS*_noAPOE_* | All available SNPs at each pT excluding the *APOE* region | 5x10^−8^ | 20 | 0.01 | 0.45 | 1.68  (-2.71-6.06) |
|  |  | 1x10^−5^ | 68 | 0.006 | 0.96 | -0.13  (-.06-4.79) |
|  |  | 0.1 | 77,268 | 0.006 | 0.92 | 0.24  (-4.43-4.91) |
| PRS*_APOEonly_* | All available SNPs within the *APOE* region at each pT | 5x10^−8^ | 46 | 0.03 | 0.13 | 3.69  (-1.13-8.51) |
|  |  | 1x10^−5^ | 62 | 0.03 | 0.16 | 3.47  (-1.40-8.33) |
|  |  | 0.1 | 118 | 0.03 | 0.13 | 3.72  (-1.16-8.60) |

**Supplementary Table 2.** Model comparisons between Receiver Operating Characteristic Areas Under the Curve using the DeLong method.

| **Model comparisons** | ***Z*-value** | ***p*-value** |
| --- | --- | --- |
| Age + Sex, Age + Sex + *APOE* status (yes/no) | -1.12 | 0.26 |
| Age + Sex, Age + Sex + *APOE_ε_*_2+_*_ε_*_4_ | -1.01 | 0.31 |
| Age + Sex, Age + Sex + PRS*_noAPOE_*+*APOE_ε_*_2+_*_ε_*_4_ | -1.83 | 0.08 |
| Age + Sex + *APOE* status (yes/no), Age + Sex + *APOE_ε_*_2+_*_ε_*_4_ | 0.67 | 0.50 |
| Age + Sex + *APOE* status (yes/no), Age + Sex + PRS*_noAPOE_*+*APOE_ε_*_2+_*_ε_*_4_ | -0.61 | 0.54 |
| Age + Sex + *APOE_ε_*_2+_*_ε_*_4_, Age + Sex + PRS*_noAPOE_*+*APOE_ε_*_2+_*_ε_*_4_ | -0.91 | 0.36 |

**Supplementary Table 3.** Regression results for the significant PRS with cognitive decline or grey matter atrophy. Raw values are shown. *APOE* SNPs: rs429358, rs7412. Baseline age, sex and PCs 1-3 included as covariates. N = 90.

| **Phenotype** | **Score** | **Score description** | **pT** | **Number**  **of SNPs** | **Adjusted R^2^** | **PRS**  ***p*-value** | **PRS β**  **(95% CI)** |
| --- | --- | --- | --- | --- | --- | --- | --- |
| Cognitive decline | PRS*_noAPOE_*+*APOE_ε_*_2+_*_ε_*_4_ | All available SNPs at each pT excluding the *APOE* region, plus the weighted sum of the two major *APOE* SNPs | 5x10^−8^ | 22 | 0.08 | 0.59 | -0.03  (-0.15-0.08) |
|  | *APOE_ε_*_2+_*_ε_*_4_ | Weighted sum of the two major *APOE* SNPs |  | 2 | 0.08 | 0.73 | -0.02  (-0.14-0.10) |
| Grey matter atrophy | PRS*_noAPOE_*+*APOE_ε_*_2+_*_ε_*_4_ | All available SNPs at each pT excluding the *APOE* region, plus the weighted sum of the two major *APOE* SNPs | 5x10^−8^ | 22 | 0.002 | 0.73 | -0.26  (-1.78-1.25) |
|  | *APOE_ε_*_2+_*_ε_*_4_ | Weighted sum of the two major *APOE* SNPs |  | 2 | 3.84x10^-5^ | 0.98 | 0.02  (-1.53-1.57) |
